# An update on the occurrence of flies (Diptera: Muscidae, Calliphoridae) and sucking lice (Phthiraptera: Anoplura) of veterinary importance in Malta: First record of *Lucilia cuprina* and *Linognathus africanus*

**DOI:** 10.3389/fvets.2023.1143800

**Published:** 2023-03-14

**Authors:** Sándor Hornok, Andrea M. Cini Bruno, Nóra Takács, Gergő Keve, Attila D. Sándor, Jenő Kontschán

**Affiliations:** ^1^Department of Parasitology and Zoology, University of Veterinary Medicine, Budapest, Hungary; ^2^ELKH-ÁTE Climate Change: New Blood-Sucking Parasites and Vector-Borne Pathogens Research Group, Budapest, Hungary; ^3^Department of Parasitology and Parasitic Diseases, University of Agricultural Sciences and Veterinary Medicine, Cluj-Napoca, Romania; ^4^Centre for Agricultural Research, Plant Protection Institute, ELKH, Budapest, Hungary; ^5^Department of Plant Sciences, Albert Kázmér Faculty of Mosonmagyaróvár, Széchenyi István University, Mosonmagyaróvár, Hungary

**Keywords:** house fly, stable fly, blowfly, myiasis, blood-sucking lice

## Abstract

To obtain new data on the species diversity, host associations and spatiotemporal occurrence of flies and blood-sucking lice of veterinary importance in Malta, ectoparasites were collected at cattle, sheep and goat, pig farms, as well as dog shelters, and in two places in the absence of domestic animals. The species were identified morphologically, but voucher specimens were also analyzed with molecular-phylogenetic methods following DNA extraction. Altogether 3,095 flies (Diptera: Muscidae, Calliphoridae) were collected at farms and kennels near domestic animals, as well as 37 blowflies (Calliphoridae) in rural and urban areas without animals nearby. Regarding Muscidae, the great majority of flies (*n* = 3,084) were identified as the common housefly (*Musca domestica*). Eight flies represented the stable fly (*Stomoxys calcitrans*). Three blowflies associated with dogs and small ruminants belonged to *Lucilia cuprina*. By contrast, all 37 blowflies collected without domestic animals nearby, were identified as *Lucilia sericata*. In addition, 22 sucking lice were collected from goats, and all belonged to *Linognathus africanus*. Molecular identification of 28 flies and four lice confirmed the above species. Considering the sex ratio of *M. domestica* among samples collected randomly at cattle farms, females predominated in the whole study period, but the abundance of males increased significantly toward the autumn. *Stomoxys calcitrans* was associated with cattle and dogs, whereas *L. cuprina* was found near small ruminants and dogs. To our knowledge, this is the first study including the molecular analysis of flies and lice of veterinary-medical importance from Malta. The most important finding of this study is the first evidence for the autochthonous occurrence of *L. cuprina* in Malta. The exclusive presence of *L. cuprina* at animal-keeping facilities in rural areas and association of *L. sericata* with urban areas void of livestock might reflect similar habitat preference of these species in Malta to what was reported in South Africa. Based on the sucking-louse burden in the examined goat herds, the situation in Malta was similar to northern Africa where the exclusive presence of *L. africanus* was reported, unlike toward the north in the Mediterranean Basin where populations of this species are mixed with *Linognathus stenopsis*.

## Introduction

Malta consists of three larger and several smaller islands that are situated in the middle of the Mediterranean region south of Italy, between Africa and Europe (1). This geographical position makes these islands very important from the veterinary-medical point of view, both economically (2) and ecologically. Considering the latter, Malta is an important stop-over site for migratory birds, flying in the autumn from Europe to their wintering grounds in Africa, then returning from Africa to Europe in the spring. Thus, during migratory activity as a natural phenomenon birds transport epidemiologically important tick species via Malta intercontinentally (3). However, Africa and Europe are also connected through these islands as a consequence of human activity, for instance during trading and transportation of livestock and pet animals.

During the past few years several new tick species (Acari: Ixodidae, Argasidae) were discovered in Malta, in part probably as a consequence of animal transportation (4). Among anopluran lice, three species of medical importance (*Pediculus humanus capitis, Pediculus humanus corporis, Phthirus pubis*) were reported from these islands (5), but data on the louse-infestation of domestic animals are lacking. Although the Diptera fauna of Malta has been extensively reviewed recently (6), it is not certain whether all muscoid and myiasis-causing flies have been surveyed on these islands or there are species that might still be indigenous but not yet found. For example, *Wohlfahrtia magnifica* (Diptera: Sarcophagidae) is known to occur in Sicily (7) <200 km from Malta. However, this fly species has never been reported in Malta (6).

Thus, the aim of this study was to compensate for the lack of updated information on ectoparasites associated with domestic (mainly livestock) animals in Malta, with special emphasis on muscoid flies (Diptera: Muscidae), myiasis-causing flies (Diptera: Sarcophagidae, Calliphoridae) and blood-sucking lice (Anoplura: Haematopinidae, Linognathidae). It was also within the scope of this study to analyze collected flies and lice with molecular biological tools, to our knowledge for the first time in the context of Malta.

## Materials and methods

### Sample collection, morphological identification

**Figure 1:**
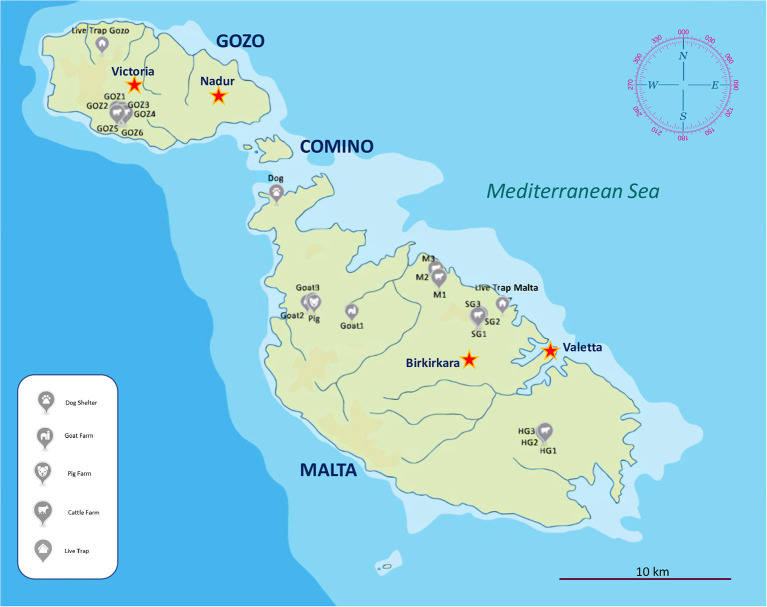
Map of the Maltese Islands indicating sampling locations (registration numbers), with the silhouette of sampled animals.

During the summer and autumn of 2020 and 2021, ectoparasites were collected on repeated (mostly monthly) occasions in rural areas: at four cattle farms, three sheep and goat farms, as well as a pig farm and two dog shelters (Figure 1, Table 1). Sampling took place between 9 a.m. and 11 a.m. Flies were caught with fish nets from the walls and gates of stables and pens below 1 m height (e.g., where the animals put through their head to feed). Samples were also collected besides the calves in a similar manner. In case of dogs the area outside the kennel cages was sampled. For comparison, after evaluating the species of blowflies caught with fish net near livestock animals at farms in July and August, 2020-2021, 37 blowflies were collected at a rural site (void of farms) in the northern part of Gozo and at an urban site in Swieqi in Malta in July, 2022. Bait traps made of plastic bottles (1.5 l bottle, upper third cut and inserted into the bottom upside down, containing pieces of decomposing chicken meat and pork) were used for this purpose. In addition, lice were removed with soft tweezers.

**Table 1A:** Flies (M: males, F: females) collected in pens and stables at cattle farms.

**Location (code)**	**Month (year)**	***Musca domestica***	***Stomoxys calcitrans***	***Lucilia cuprina***
Maghtab (MH)	July (2020)	45M + 123F	−	−
	August (2021)	87M + 131F	−	−
	September (2021)	366M + 318F	−	−
Hal' Ghaxaq (HG)	July (2020)	116M + 228F	1M	−
San Gwann (SG)	July (2020)	27M + 199F	−	−
	October (2020)	63M + 109F	−	−
	November (2020)	54M + 101F	−	−
Sannat (GO)	July (2020)	94M + 357F	1F	−
**Total** **=**		**2418**	**2**	−

The location is also shown on the map (Figure 1) according to the code in parentheses.

**Table 1B:** Flies (M: males, F: females) collected near goats/sheep, pigs or dogs.

**Animals kept (code or location)**	**Month (year)**	***Musca domestica***	***Stomoxys calcitrans***	***Lucilia cuprina***
Goats, sheep (1)	July (2021)	20M + 67F	−	−
	August (2021)	26M + 63F	−	−
	September (2021)	13M + 35F	−	−
Goats, sheep (2)	July (2021)	26M + 136F	−	1M + 1F
	August (2021)	20M + 18F	−	−
	September (2021)	26M + 40F	−	−
Sheep (Maghtab)	September (2021)	11M + 8F	−	−
Pigs (Mgarr)	August (2021)	3M + 2F	−	−
	September (2021)	8M + 5F	−	−
Dogs (Manikata)	August (2020)	15M + 25F	−	1F
Dogs (Noah's ark)	July (2021)	3M + 36F	1F	−
	August (2021)	16M + 28F	−	−
	September (2021)	7M + 9F	5M	−
**Total** **=**		**666**	**6**	**3**

The location is also shown on the map (Figure 1).

All flies and lice were stored in 96% ethanol, and their species/sexes were morphologically identified according to standard keys (8–11). Pictures of representative specimens were made with a VHX-5000 digital microscope (Keyence Co., Osaka, Japan).

### DNA extraction

Molecular identification of selected flies was performed as outlined below (all individuals from the genus *Stomoxys*, 17 *Lucilia*, three *Musca* and four *Linognathus* specimens). DNA was extracted from two legs of 28 flies and four lice that served as voucher for species identification, with the QIAamp DNA Mini Kit (QIAGEN, Hilden, Germany) according to the manufacturer's instruction, including an overnight digestion in tissue lysis buffer and Proteinase-K at 56°C, as reported (3, 4). An extraction control was also processed in each set of samples.

### Molecular analyses of flies and blood-sucking lice for species identification

A 710 bp-long fragment of the cox1 (cytochrome c oxidase subunit I) gene was amplified from fly DNA extracts with a conventional PCR modified from Folmer et al. (12). The primers HCO2198 (5′-TAA ACT TCA GGG TGA CCA AAA AAT CA-3′) and LCO1490 (5′-GGT CAA CAA ATC ATA AAG ATA TTG G-3′) were used in a reaction volume of 25 μl, containing 1 U (0.2 μl) HotStarTaq Plus DNA polymerase, 2.5 μl 10x CoralLoad Reaction buffer (including 15 mM MgCl_2_), 0.5 μl PCR nucleotide Mix (0.2 mM each), 0.5 μl (1 μM final concentration) of each primer, 15.8 μl ddH_2_O and 5 μl template DNA. During the amplification, the initial denaturation step at 95°C for 5 min was followed by 40 cycles of denaturation at 94°C for 40 s, annealing at 48°C for 1 min and extension at 72°C for 1 min. Final extension was performed at 72°C for 10 min.

For the identification of blood-sucking lice another PCR was used, which amplifies an approx. 420 bp-long-fragment of the cox1 gene of Phthiraptera with the primers L6625 (5′-CCG GAT CCT TYT GRT TYT TYG GNC AYC C-3′) and H7005 (5′-CCG GAT CCA CNA CRT ART ANG TRT CRT G-3′) (13). During the amplification, the initial denaturation step at 95°C for 5 min was followed by 4 cycles of denaturation at 95°C for 1 min, annealing at 45°C for 1 min and extension at 72°C for 1 min then 30 cycles of denaturation at 95°C for 1 min, annealing at 61°C for 1 min and extension at 72°C for 1 min. Final extension was performed at 72°C for 7 min.

### PCR controls, sequencing and phylogenetic analyses

PCRs were run with appropriate sequence-verified positive controls and non-template reaction mixture as a negative control. Extraction controls and negative controls remained PCR negative. Purification and sequencing of the PCR products were done by Biomi Ltd. (Gödöllo, Hungary). Sequences were checked with the BioEdit program (https://bioedit.software.informer.com/7.2/), then compared to GenBank data by nucleotide BLASTN program (https://blast.ncbi.nlm.nih.gov). Representative sequences are available under accession numbers OP579106-OP579114 (flies) and OP658950 (louse). Sequences from other studies (retrieved from GenBank) were included in the phylogenetic analyses only if they had nearly 100% coverage with sequences from this study. This dataset was resampled 1,000 times to generate bootstrap values. Phylogenetic analyses were conducted with the Maximum Likelihood method, Jukes-Cantor model by using MEGA version 7.0 (14).

### Statistical analysis

Abundance (i.e., the number of flies in a group *vs* all other flies) was analyzed by Fisher's exact test (https://www.langsrud.com/fisher.htm). Differences were regarded as significant if *P* < 0.05.

## Results

### Morphological identification of species

**Figure 2:**
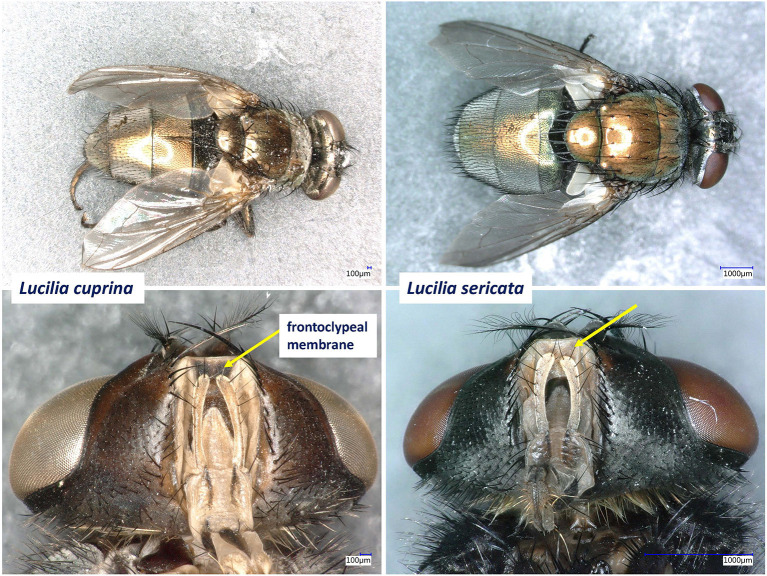
Habitus and frontoclypeal membrane of *Lucilia cupina* sampled in Malta **(Left)**, in comparison with *L. sericata* **(Right)**. The yellow arrows indicate the frontoclypeal membrane.

Altogether, 3,095 flies (Diptera: Muscidae, Calliphoridae) were collected at farms and kennels near domestic animals and 37 blowflies (Calliphoridae) in rural (*n* = 4) and urban (*n* = 33) areas without animals nearby. Regarding Muscidae, the great majority of flies (99.6%: *n* = 3084) were identified as the common housefly *Musca domestica*, based on the position of eyes (wide frons), shape of the abdomen (longitudinally flattened, pointed at the end) and setation of the propleuron. Eight flies (0.3%) represented the stable fly *Stomoxys calcitrans* based on the forward projecting proboscis and dotted abdominal pattern. In addition, three blowflies (0.1%) collected near animals belonged to *Lucilia cuprina* based on the dark color of the frontoclypeal membrane (Figure 2) and the number of setae on the scutellum. The 37 blowflies collected with bait traps, without domestic animals nearby, were identified as *L. sericata*.

Sucking lice (*n* = 22) were only found on 20 goats at three farms. All these belonged to *Linognathus africanus*, based on the semicircular, sclerotized ocular elevation behind the antennae and the structure of the last abdominal segment (Supplementary Figure 1).

### Molecular-phylogenetic identification of species

In general, molecular identification confirmed the results of morphological identification. Among the flies, three *M. domestica* had two cox1 haplotypes, one with 100% (636/636 bp) sequence identity to specimens reported from both the Old and the New Worlds, e.g., from the UK (GenBank: HE614023). The other *M. domestica* cox1 sequence (OP579114) was new, with up to 99.8% (635/636 bp) maximum identity to any other sequence of this species available in GenBank.

**Figure 3:**
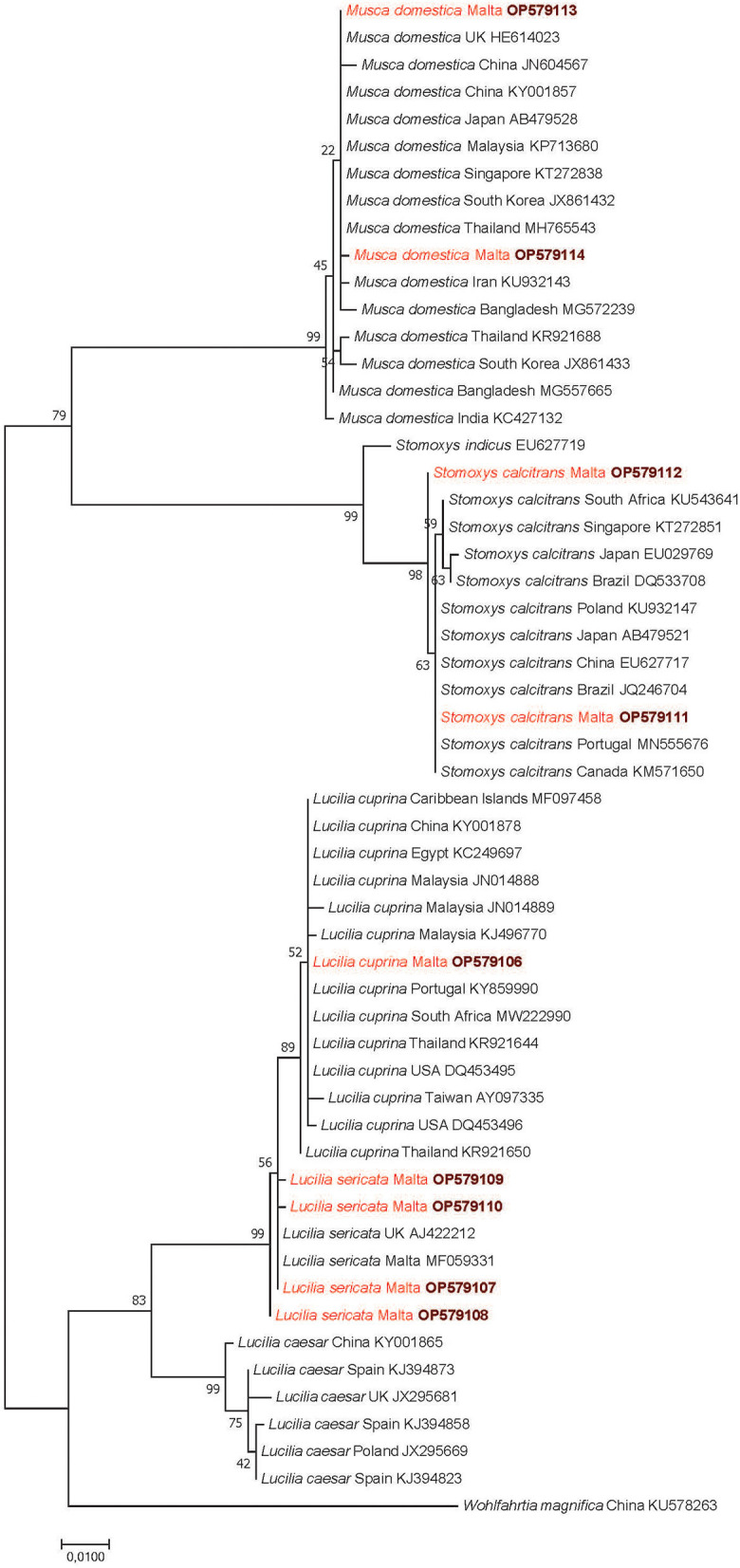
Phylogenetic tree of Diptera based on the cox1 gene, made with the Maximum Likelihood method and the Jukes-Cantor model. In each row, after the species or genus name, the country of origin and the GenBank accession number are shown. Sequences obtained in this study are in red and bold accession numbers. The analysis involved 56 nucleotide sequences and 1000 bootstrap replications. There were a total of 631 positions in the final dataset. The scale-bar indicates the number of substitutions per site.

Similarly, the majority (7 of 8) of *S. calcitrans* cox1 sequences were 100% (636/636) identical to several sequences in GenBank (e.g., from Canada: KM571650, Brazil: JQ246704, Portugal: MN555676), but one haplotype (OP579112) was new, with a maximum of 99.8% (635/636 bp) identity to the closest matches in GenBank. All three *L. cuprina* from Malta had cox1 sequences 100% (636/636 bp) identical to *L. cuprina* from South Africa (MW222990) and Portugal (KY859990). *Lucilia sericata* individuals that were analyzed for the cox1 gene (n = 14) belonged to four haplotypes, with 99.8-100% (635–636/636 bp) identity to a sequence of this species deposited in GenBank from Malta (MF059331). Phylogenetic analyses showed that the cox1 sequences of all flies collected on the Maltese islands clustered with conspecific flies (Figure 3).

The cox1 sequences of four *L. africanus* lice were identical and showed only 99.4% (358/360 bp) identity to the only sequence (EU375760) of this species available in GenBank, reported for a specimen removed from a goat.

### Observations on the host-association, sex ratio and monthly distribution of flies

Considering the sex ratio of *M. domestica* among samples collected randomly at cattle farms, females predominated in the whole study period (*n* = 1,566 vs 852 males), but the abundance of males increased significantly from July to August (*P* = 0.009) and August to September (*P* = 0.0006) at one place, and from July to October, then from October to November (*P* < 0.0001) at another place where repeated samplings were performed. On the contrary, the sex ratio of this fly species remained relatively constant in one place where small ruminants were kept, but the ratio of males increased from July to August or to September at another sampling site with sheep and goats (*P* < 0.0001 and *P* = 0.0002, respectively). This phenomenon was also observed at the dog kennel (*P* = 0.003 and 0.004, respectively) (Table 1).

## Discussion

To our knowledge, this is the first study including the molecular analysis of flies and lice of veterinary-medical importance in Malta. Regarding muscoid flies, the common housefly, *M. domestica* is a fly species known for its epidemiological role as a carrier of pathogens (15), and significant economic losses can be attributed to its presence and the resultant annoyance (16). It is not surprising that *M. domestica* was found to be the most abundant fly species in Malta, taking into account previous data on its occurrence in this country (17).

The stable fly, *S. calcitrans* has similarly high veterinary-medical significance, owing to its aggressive, persistent blood-feeding behavior and potential vector role affecting both domestic animals and humans (18). This muscoid fly species has also been long known to occur in Malta, where its breeding places are limited (17), explaining their low number in the present study. Finding of exclusively this cosmopolitan *Stomoxys* species suggests the absence of the African *Stomoxys niger* from the Dipteran fauna of Malta, as already reported (6). The majority of *S. calcitrans* cox1 haplotypes from Malta were identical, reflecting low genetic diversity. This may be in part related to reproductive isolation of stable fly populations in Malta from those in mainland Europe, probably because they are not readily transported with livestock or canine hosts and their flight range is within 30 km (19).

Among calliphorid flies, *L. cuprina* is a very important causative agent of fly strike (myiasis) particularly affecting sheep and other livestock, but potentially all warm-blooded vertebrates (20). Perhaps, the most important finding of this study is the first evidence for the autochthonous occurrence of *L. cuprina* in Malta where even recently it has not been known to occur (6). This blowfly species most likely started its geographic range expansion from Australia and South Africa (21, 22). The cox1 haplotype identified in Malta clustered in the phylogenetic group including sequences of *L. cuprina* from Portugal and South Africa, in line with this geographical origin.

In Europe, *L. cuprina* has already been reported from Spain (23), Turkey [and the Middle East: (24)], and on single occasions from other countries [Czech Republic: (25); southern Ukraine: (26); UK: (27)]. Interestingly, this blowfly species is not known to occur in Italy (i.e., directly north of Malta), because when it was reported in that country, it turned out to be a misidentification (28). Owing to the preference of *L. cuprina* for warmer climate (20), it is unlikely that it will establish toward the north, but consequent to its emergence in several parts of southern Europe, probably it will become widespread in the Mediterranean Basin.

While *L. cuprina* was exclusively found near domestic animals in Malta, in other types of rural and urban locations only *L. sericata* could be collected with bait traps. This is unlikely a consequence of different collection methods, because both of these species are attracted to baits (29), and both are attracted to ovine hosts and their responses to chemical stimuli are broadly similar (30). Rather, this maybe related to differences in their habitat preference, similarly to what was reported in South Africa, where *L. sericata* occurs in urban areas and is not found in rural settings (31, 32).

Regarding host associations, *M. domestica* was reported to predominate near pigs in Malta (17) but in this study it showed very high abundance near cattle, small ruminants and dogs. The present results also confirmed previous data that *S. calcitrans* can be found mainly on or around cattle in Malta (17), but during our study this fly species was also collected near dogs, as reported in other countries and justifying its other name as dog fly (33). *Lucilia cuprina* was found near dogs and small ruminants in Malta, i.e., close to animals in which it can cause myiasis (20, 34), justifying the need for further evaluation of the country-wide veterinary significance of this species.

In this study, *M. domestica* was found to be the most abundant fly species at farms during the summer and autumn. This is in line with previous reports from Malta on the activity of this species, as it was observed between April and November (17).

Concerning sex ratio in the context of seasonality, it was reported that males and females of *M. domestica* are different in their physical activity and mating behavior (35). In addition, males and females mature at different ages (36, 37). Therefore, in populations of *M. domestica* different sex-ratios and densities can be expected to occur under natural conditions (38). For instance, predominance of females was reported in other studies (39, 40) similarly to the results shown here. This was modified toward the autumn when the shift toward a higher ratio of males might reflect decreasing activity which is in favor of their longer survival (41). The sex ratio of *M. domestica* is crucial to know properly, because it will influence locomotor activity (38), feeding behavior and thus the role in pathogen transmission, as well as the sensitivity to biological means of control (16). At the same time, the individual number of *S. calcitrans* was too low in this study to assess seasonality.

The sucking louse species *L. africanus* was already reported in certain regions of the Mediterranean Basin, as exemplified by Sardinia (42) but recently also in one case in the Balkans, in Bulgaria (43). According to the results of this study, it is also established in Malta. Based on the three examined goat herds, the situation was similar to northern Africa (Algeria) where the exclusive presence of *L. africanus* was reported (44), unlike north of Malta in the Mediterranean Basin (Sardinia) where populations of this species are mixed with *L. stenopsis* (42).

## Data availability statement

The datasets presented in this study can be found in online repositories. The names of the repository/repositories and accession number(s) can be found in the article/Supplementary material.

## Author contributions

SH: conceptualization, study design, fly identification, DNA extraction, and manuscript writing. AC: sample collection, fly identification, data curation, and manuscript writing. NT: PCR tests and sequencing. GK: study design and manuscript writing. ADS: sample collection. JK: phylogenetic analyses and digital photography. All authors contributed to the article and approved the submitted version.

## Supplementary material

Supplementary Figure 1 Ventral view of *Linognathus africanus* male and female, collected in Malta.
