# Supplementary figures and images for: An update on the occurrence of flies (Diptera: Muscidae, Calliphoridae) and sucking lice (Phthiraptera: Anoplura) of veterinary importance in Malta: First record of Lucilia cuprina and Linognathus africanus
Source: Front Vet Sci. 2023 Mar 14;10:1143800. doi: 10.3389/fvets.2023.1143800 (PMC10043312; doi:10.3389/fvets.2023.1143800)

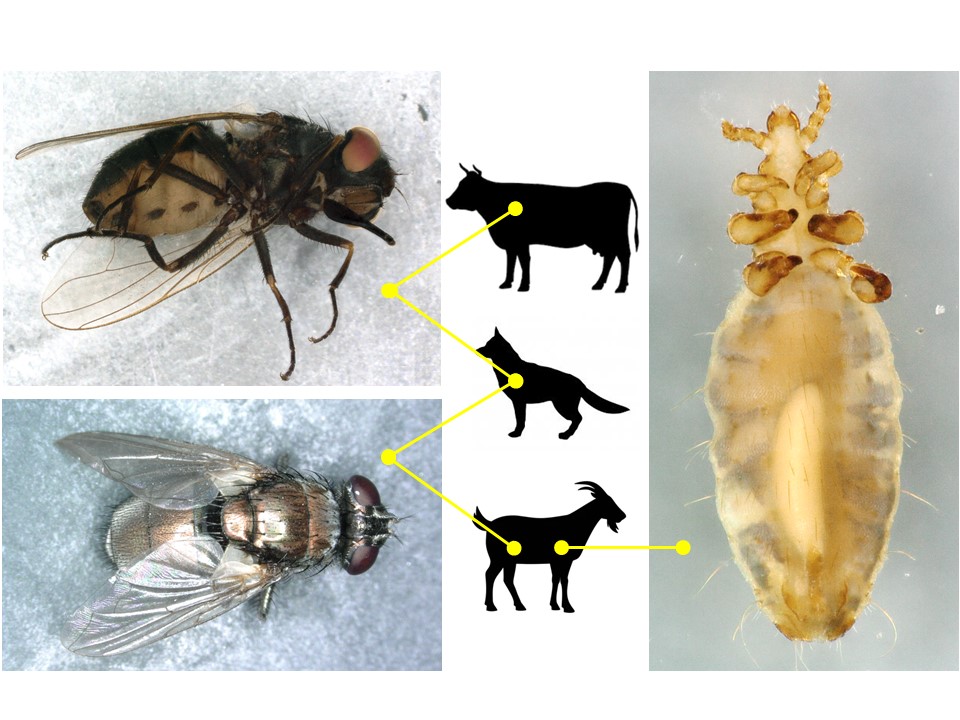

Supplement: Supplementary Figure 1 — Ventral view of Linognathus africanus male and female, collected in Malta. [file Image_1.JPEG]

*Linognathus africanus*

100µm

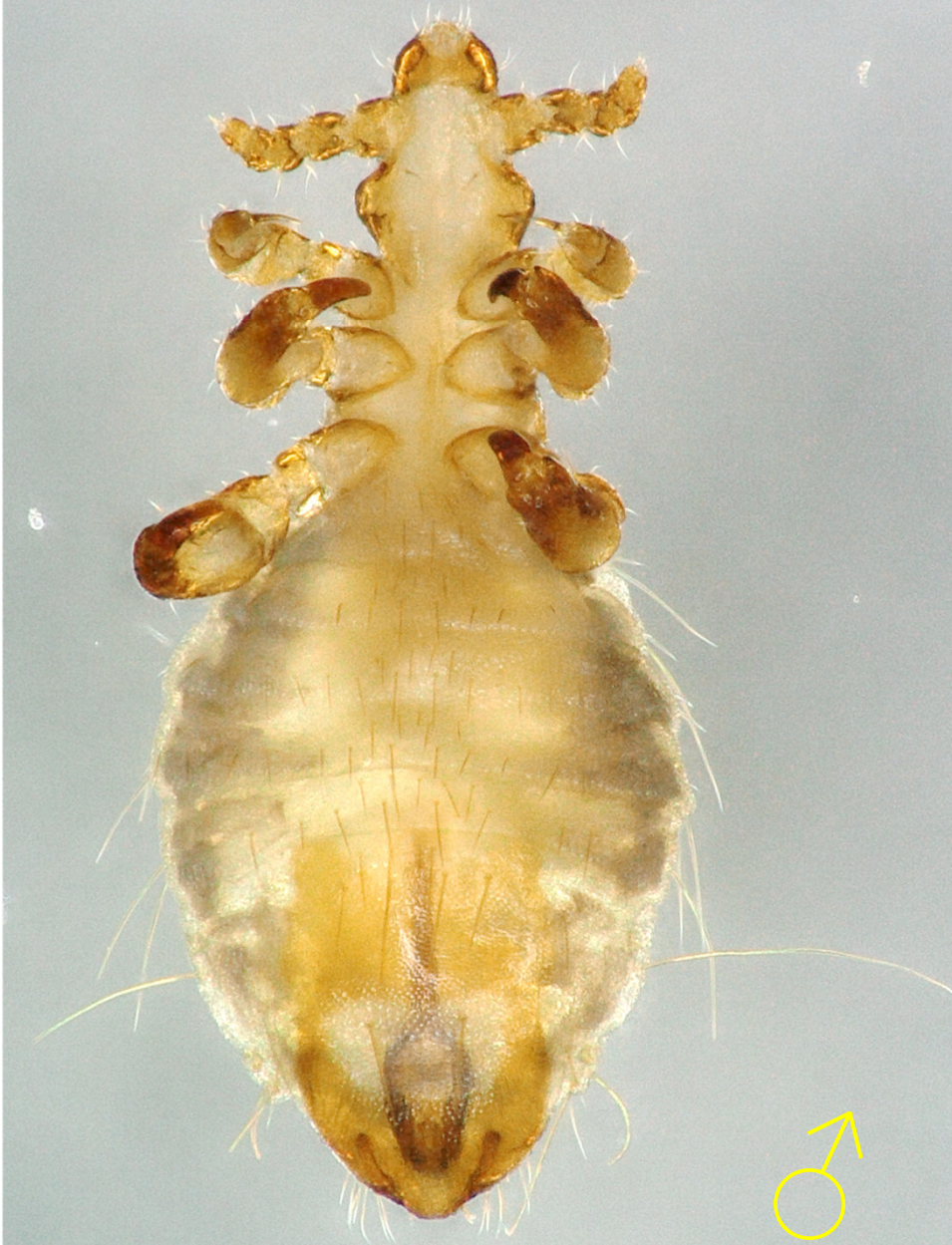

100µm

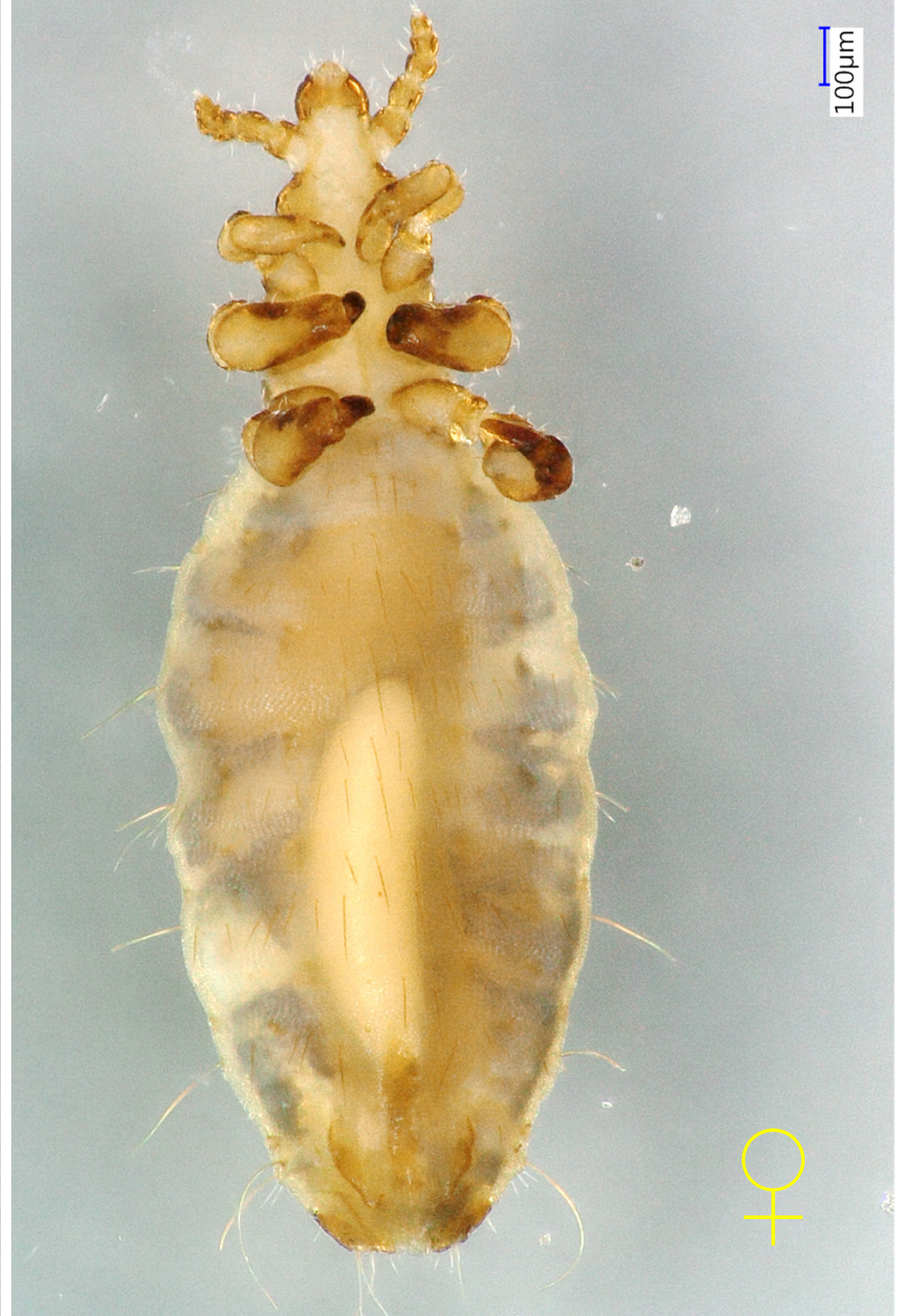

Supplement: Supplementary file 2 [file Data_Sheet_1.PDF]
